# IFN-γ downregulates miR-4319 to enhance NLRC5 and MHC-I expression in MHC-I-deficient breast cancer cells

**DOI:** 10.1080/15384047.2025.2523621

**Published:** 2025-07-01

**Authors:** Ming-Zhen Zhao, Hua-Chuan Zheng, Yu Sun, Xiao-Feng Jiang, Li Liu, Chun-Yan Dang, Jun-Ying Li, Li-Xin Sun

**Affiliations:** a Hebei Key Laboratory of Panvascular Diseases, The Affiliated Hospital of Chengde Medical University, Chengde, Hebei, China; b Department of Central Laboratory, The Affiliated Hospital of Chengde Medical University, Chengde, Hebei, China; c Department of Spine Surgery, The Affiliated Hospital of Chengde Medical University, Chengde, Hebei, China; d Department of Rehabilitation, The Affiliated Hospital of Chengde Medical University, Chengde, Hebei, China; e Department of Oncology, The Affiliated Hospital of Chengde Medical University, Chengde, Hebei, China; f Department of Infectious Diseases, Maternal and Child Health Hospital of Pingquan City, Pingquan, Hebei, China

**Keywords:** Breast cancer, miR-4319, NLRC5, MHC class I, immunotherapy

## Abstract

Sufficient MHC-I expression on cancer cells is essential for the recognition and killing of cancer cells by immune effector cytotoxic T-lymphocyte (CTL). An important mechanism of cancer immune escape is loss or down-regulation of MHC-I. This is frequently associated with reduced expression of NOD-like receptor (NLR) caspase recruitment domain containing protein 5 (NLRC5), genetically and epigenetically. NLRC5, a regulator of MHC-I, has been identified as a potential target of miR-4319 due to its complementary binding site for miR-4319, according to prediction by TargetScan (http://www.targetscan.org/). Inhibition of miR-4319 by IFN-γ (known as MHC-I increasing agent) to upregulate NLRC5 with upregulation of MHC-I in MHC-I-deficient breast cancer cells, however, remains unclear. After treatment with IFN-γ, miR-4319 was detected with qRT-PCR; NLRC5 protein was detected with western-blot; and MHC-I mRNA and protein were detected with qRT-PCR and western-blot, respectively. It was found statistically that miR-4319 was lower and NLRC5 protein was higher in groups of 50 U/ml and 100 U/ml IFN-γ, and MHC-I mRNA and protein were higher in all groups of different concentrations of IFN-γ, except for HLA-A protein in 25 U/ml IFN-γ group, with dose dependent tendency, compared with the control group. IFN-γ inhibits miR-4319 and upregulates NLRC5, thereby enhancing expression of MHC-I in SKBR3 breast cancer cells, while limitations include the absence of functional rescue experiments and in vivo validation. Along with direct cytotoxicity on tumor cells, IFN-γ’s immunomodulatory effect strengthens tumor immunogenicity, counteracts immune evasion mechanisms, and potentially improves the efficacy of cancer immunotherapy.

## Introduction

Although numerous efforts have been made for the prevention and control of cancer, it remains a major health problem worldwide. Breast cancer is one of the most common cancers in women as well as one of the most common cancers in both sexes combined. It was estimated that there were 19.3 million new cancer cases and almost 10.0 million cancer deaths worldwide in 2020.^1^ In these new cancer cases, female breast cancer cases were 2.3 million which surpassed lung cancer and became the most commonly diagnosed cancer. What’s worse, the new death cases of breast cancer were 0.7 million, which became the first leading cause of cancer death in women and the fifth leading cause of cancer death in both sexes combined. Development of cancer including breast cancer is associated with both genetic and non-genetic risk factors. The host immune system may protect the host from malignancy. But the immune evasion has been found in many cancer cells including breast cancer cells, whereby not only the host immune system may fail to protect the host but also the immunotherapy may be impeded. Loss or down-regulation of major histocompatibility complex (MHC) class I is an important mechanism of immune evasion in cancer cells.

Recently, MiR-4319 has been found as a tumor suppressor in several human cancers.^2^ According to prediction by TargetScan (http://www.targetscan.org/), NOD-like receptor (NLR) caspase recruitment domain containing protein 5 (NLRC5) has a complementary binding site for miR-4319, therefore being identified as a potential target of miR-4319.^2^ NLRC5 is a regulator of MHC class I transcription.^3^ MHC class I is also known as human leukocyte antigen (HLA) class I in human. IFN-γ is known as MHC class I increasing agent. In terms of promotion of MHC class I expression, the regulatory role of IFN-γ in suppressing miR-4319 and upregulating NLRC5 in MHC-I-deficient breast cancer is considerable and remains unclear.

The host immune system plays a central and complex role in immunosurveillance. The immunosurveillance constantly protects the host against foreign threats (e.g. infections) and damaged cells undergoing stress or malignant transformation.^4^ Accordingly, cancer immunotherapy has been developed and has become a powerful clinical strategy for cancer therapy and is recognized as a promising strategy to treat and even cure certain types of cancer.^5,6^ In immunotherapy, agents are used to activate or boost the activation of the immune system to combat cancer cells through natural mechanisms. For breast cancer, multiple trials with breast cancer vaccines to deliver shared tumor antigens have demonstrated safe and inducible antigen-specific immune responses.^7^ Promising effects of immune checkpoint inhibitors have been shown with the potential of harnessing the immune system for clinical benefit.^7^ Checkpoint inhibitors target cytotoxic T-lymphocyte-associated protein 4 (CTLA-4) and the programmed cell death protein-1 (PD-1)/programmed cell death ligand-1 (PD-L1).

The capacity of the immune system to eradicate the majority of arising tumors, or even control advanced ones, depends on activation of effector responses. For activation of the effector responses, specific tumor antigens are presented by MHC I molecules, and recognized by CD8+ T cells, resulting in the secretion of cytotoxic molecules and effector cytokines to kill the target cells.^8^ The loss or down-regulation of MHC class I associates with progression of the disease, level of tumor-infiltrating lymphocytes (TIL), and overall survival.^9–14^ Several groups have reported that impairment on MHC class I antigen processing and presentation is a predictor of (acquired) resistance to immune checkpoint inhibitors therapy^15–17^ and adoptive cell therapy.^18^

NLRC5 plays an important role in regulation of MHC class I expression, and therefore constitutes a target for cancer immune evasion. Reduced expression of MHC class I and related genes in cancers including breast cancer is frequently associated with genetic and epigenetic reduction of NLRC5 expression. This MHC class I reduction association with NLRC5 reduction relates to impaired CD8+ T-cell activation and poor patient prognosis.^19^ Upregulation of NLRC5 with promotion on MHC class I expression affiliates host immune system to combat cancer immune evasion, and contribute to cancer immunotherapy, including immune checkpoint inhibitors.

Dual-luciferase activity reporter assay demonstrated the direct binding of miR-4319 with the 3’-untranslated region (UTR) of NLRC5. Overexpression of miR-4319 in both KYSE150 and EC9706 cells inhibited NLRC5 expression. Based on these data, NLRC5 has been validated to be a direct downstream target of miR-4319.^2^ In addition to the role of miR-4319 in tumor-suppression, miR-4319 inhibition could be a potential strategy to enhance NLRC5 expression, thereby promoting MHC class I expression, which is critical in cancer immunotherapy, including PD1/PD-L1 blockade.

Interferon-γ (IFNγ) is a cytokine with direct cytotoxic effects on tumor cells.^20^ Our previous study has demonstrated that upregulation of NLRC5 by IFN-γ resulted in increasing MHC class I expression in MHC class I – deficient SKBR3 breast cancer cells.^21^ However, a crucial remaining unexplored gap is whether IFN-γ can exert its immunomodulatory effects by suppressing miR-4319, thereby facilitating NLRC5-mediated upregulation of MHC class I on cancer cells. Additionally, it remains unclear which specific subtype(s) among the three human classic MHC class I molecules (HLA-A, HLA-B, and HLA-C) are influenced in this process.

To address these critical gaps, the present study investigates the potential inhibitory effect of IFN-γ on miR-4319, its subsequent impact on NLRC5 expression, and the specific regulation of classical MHC class I subtypes in MHC class I – deficient SKBR3 breast cancer cells. By elucidating these mechanisms, this study provides new insights into the interplay between IFN-γ, miR-4319, and NLRC5, which could have significant implications for improving cancer immunotherapy strategies.

## Materials and methods

### Culture and preparation of breast cancer cells

SKBR3 cells are MHC class I deficient human breast cancer cells^22,23^ which were maintained under standard cell culture conditions and the cells at 6 passage were used to control potential passage-dependent effects.^24^ The SKBR3 cells at a density of 6 × 10^5^/well were seeded into 6-well culture plates and cultured in DMEM medium supplemented with 10% FBS at 37°C in a humidified atmosphere containing 5% CO^2^ for 24 h. Then, the cells were precultured in the medium containing 1% FBS for 12 h, followed by addition of IFN-γ to the indicated final concentration in the medium containing 10% FBS, for a further 24-h incubation.^21^ Additionally, considering metabolism of IFN-γ and influence of other materials in vivo, after preculturing SKBR3 cells in the medium containing 1% FBS for 12 h, plasma isolated from 6 to 8 weeks old female BALB/c mice (Beijing Vital River Laboratory Animal Technology Co., Ltd., Beijing, China, Certificate Number SCXK2016–0006) maintained under SPF conditions 24-h after last intravenously injection with IFN-γ (2 × 10^5^U/moue duplicate with 24-h interval)^25,26^ or PBS (control) into the mice (weight of the mice ranged from 20 to 25 g) was added into the cells (10% plasma in DMEM medium was used) and cultured for a further 24-h. Cells were harvested for assay. All experiments were individually performed three times.

### Quantitative real-time PCR

After 24-h treatment with IFN-γ or plasma isolated from BALB/c mice after injection with IFN-γ, the total RNAs in the SKBR3 cells were extracted using TRIzol reagent (Invitrogen, Carlsbad, USA), except for miR-4319 detection for which small RNAs were extracted using RNAiso reagent (Takara Bio USA, Inc., Mountain View, USA), under RNase free condition, according to manufacturer’s instruction, and were reverse transcribed into cDNAs using FastQuant RTkit (Tiangen Biotech Co., Ltd., Beijing, China) except for miR-4319 detection for which Mir-X miRNA First-Strand Synthesis Kit (Takara Bio USA, Inc.) was used. The responsible primers (all the primers used for qRT-PCR) were obtained from GeneCopoeia, NLRC-5 (GeneCopoeia Inc., Germantown, Maryland, USA), α heavy chain of HLA-A (GeneCopoeia Inc.), HLA-B (GeneCopoeia Inc.), HLA-C (GeneCopoeia Inc.), miR-4319 (Takara Bio USA, Inc.), and GAPDH (GeneCopoeia Inc.) were used in Quantitative real-time PCR (qPCR) using SuperReal PreMix Plus kit (Tiangen Biotech Co., Ltd., Beijing, China), except for miR-4319 detection for which the responsible primers, miR-4319 (Guangzhou RiboBio Co., Ltd, Guangzhou, China) and U6 (Takara Bio USA, Inc.) were used in qPCR using Mir-X miRNA qRT-PCR TB Green Kit (Takara Bio USA, Inc.), on Roche Cobas z 480 Real-Time PCR Detection System (Roche, Basel, Switzerland). Normalized by expression of GAPDH mRNA as a control (for miR-4319 detection U6 was used as control), the expression of the mRNAs was analyzed by using the 2^−ΔΔCt^ method. The sequences of the primers and their working concentrations are listed in Table 1.

**Table 1.** The sequences of the primers.

Primer	Forward Sequence	Reverse Sequence	working concentration
HLA-A	AGATACACCTGCCATGTGCAGC	GATCACAGCTCCAAGGAGAACC	0.5 μM
HLA-B	CTGCTGTGATGTGTAGGAGGAAG	GCTGTGAGAGACACATCAGAGC	0.5 μM
HLA-C	GGAGACACAGAAGTACAAGCGC	ACATCCTCTGGAGGGTGTGAGA	0.5 μM
NLRC5	AGTGGCTCTTCCGCTTGGACAT	CGGAACCCTAAGAACTTGGCTG	0.5 μM
GAPDH	GTCTCCTCTGACTTCAACAGCG	ACCACCCTGTTGCTGTAGCCAA	0.5 μM
miR-4319	not public by manufacturer for patent	not public by manufacturer for patent	0.5 μM

### Western blot

After the SKBR3 cells grown in 6-well culture plates were treated and untreated with IFN-γ or plasma isolated from BALB/c mice after injection with IFN-γ for 24 h, the total cellular proteins were extracted by lysis with radioimmunoprecipitation assay (RIPA) buffer (Beijing Solarbio Science & Technology Co., Ltd., Beijing, China) and quantified with BCA Protein Assay Kit (Beijing Solarbio Science & Technology Co., Ltd., Beijing, China). After 10% sodium dodecyl sulfate-polyacrylamide gel electrophoresis (SDS-PAGE), the total cellular proteins extracted from the SKBR3 cells were electrotransferred to polyvinylidene fluoride (PVDF) membranes. Following 2-h incubation with 5% skim milk at room temperature for blockade, the individual primary antibodies, polyclonal rabbit anti-NLRC5 antibody (Abcam, Cambridge, England), anti-HLA-A antibody (Abcam, Cambridge, England), anti-HLA-B antibody (Abcam, Cambridge, England), anti-HLA-C antibody (Abcam, Cambridge, England), anti-STAT1 antibody (Affinity Biosciences, Cincinnati, OH, USA, Cat.#: AF6300), anti-p-STAT1 antibody (Affinity Biosciences, Cincinnati, OH, USA, Cat.#: AF3300) and mouse monoclonal anti-GAPDH antibody (OriGene Technologies Inc., Rockville, USA), were added for overnight incubation at 4°C, and then after three washes with TBST, horseradish peroxidase-conjugated secondary antibodies were added for 2-h incubation. The bands were visualized with ECL and quantified using Image J software (US National Institutes of Health, Bethesda, ML, USA) with normalization by GAPDH in relative intensity manner compared with the control. The antibodies and their working concentrations are listed in Table 2.

**Table 2.** The antibodies and their working concentrations.

antibody	dilution	working concentration (µg/ml)
HLA-A	1:1000	0.205
HLA-B	1:1000	1
HLA-C	1:2000	1
NLRC5	1:1000	1
STAT1	1:1000	1
pSTAT1	1:1000	1
GAPDH	1:5000	0.2

### Statistical analysis

The data were presented as mean ± standard deviation (SD) and analyzed by SPSS software for LSD test after one-way ANOVA or t-test to determine the statistical significance. Three independent experiments (*n* = 3) were performed. *p* < .05 was statistically considered significant.

## Results

### The levels of miR-4319

**Figure 1.**
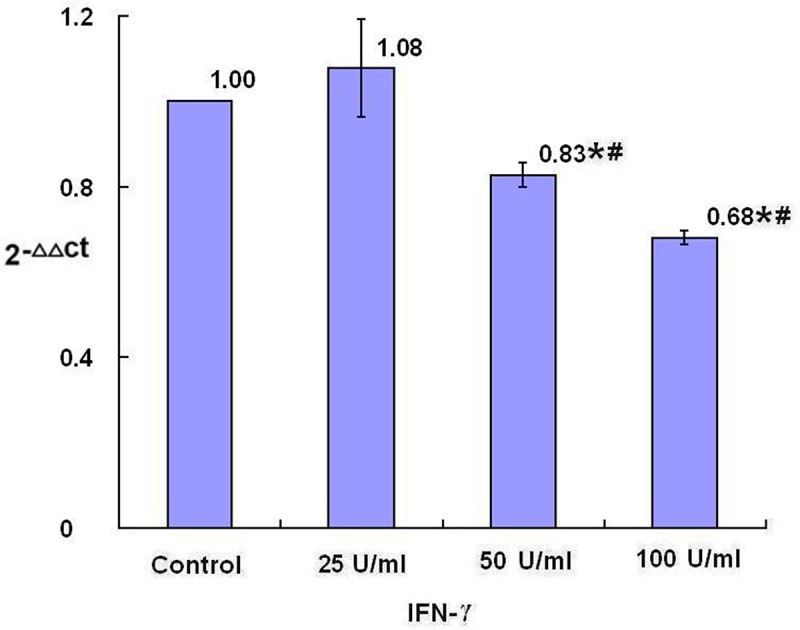
The levels of miR-4319 after treatment with IFN-γ (*n* = 3). After treatment of SKBR3 breast cancer cells with IFN-γ for 24 h, the levels of miR-4319 were detected by qRT-PCR. Error bars indicate standard deviation of the means. Asterisks indicate LSD p-values <0.05, compared with the control after one-way anova. Hashtags indicate LSD p-values <0.05, compared with the 25 U/ml IFN-γ group after one-way anova.

**Figure 2.**
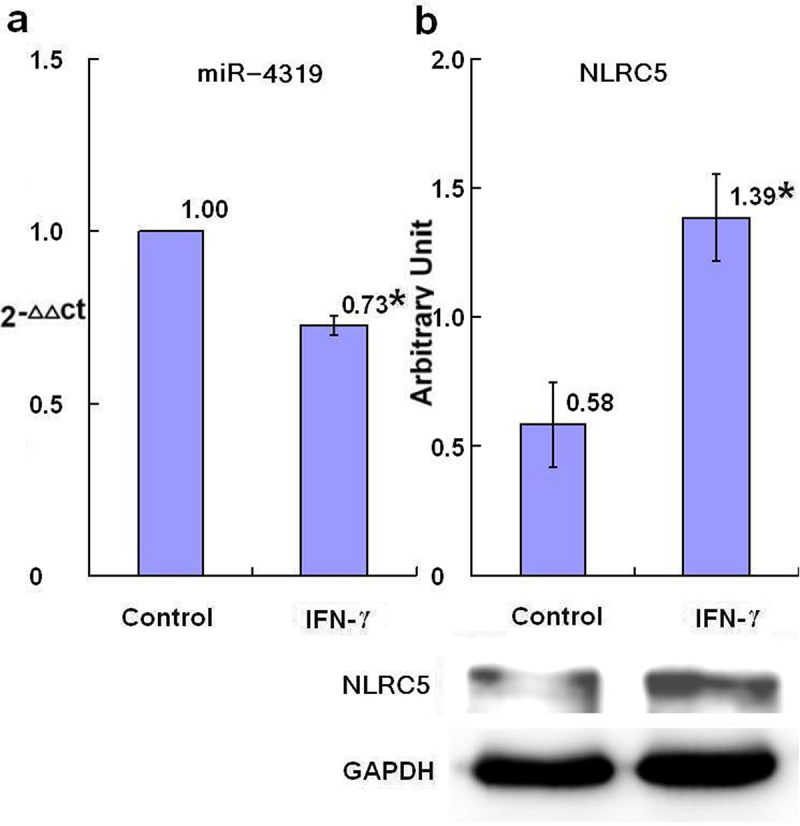
The level of miR-4319 and expression of NLRC5 protein after treatment with mice plasma (*n* = 3). After 24-h treatment of SKBR3 breast cancer cells with mice plasma isolated from BALB/c mice 24-h after injection with IFN-γ or PBS (control), the level of miR-4319 was detected by qRT-PCR (a) and expression of NLRC5 protein was detected by Western blot (b). Error bars indicate standard deviation of the means. Asterisks indicate p-value <.05, compared with the control after t-test.

The relative levels of miR-4319 in SKBR3 breast cancer cells after 24-h treatment with IFN-γ, were decreased in the groups treated with 50 U/ml and 100 U/ml IFN-γ, with dose dependent tendency compared with the control group with statistical significance (*p* < .05) (Figure 1). Treatment with plasma isolated from BALB/c mice after injection with IFN-γ for 24-h, the relative levels of miR-4319 in SKBR3 breast cancer cells were decreased as well, compared with the control group (treatment with plasma isolated from BALB/c mice after injection with PBS) with statistical significance (*p* < .05) (Figure 2a)

### The expression of NLRC5

**Figure 3.**
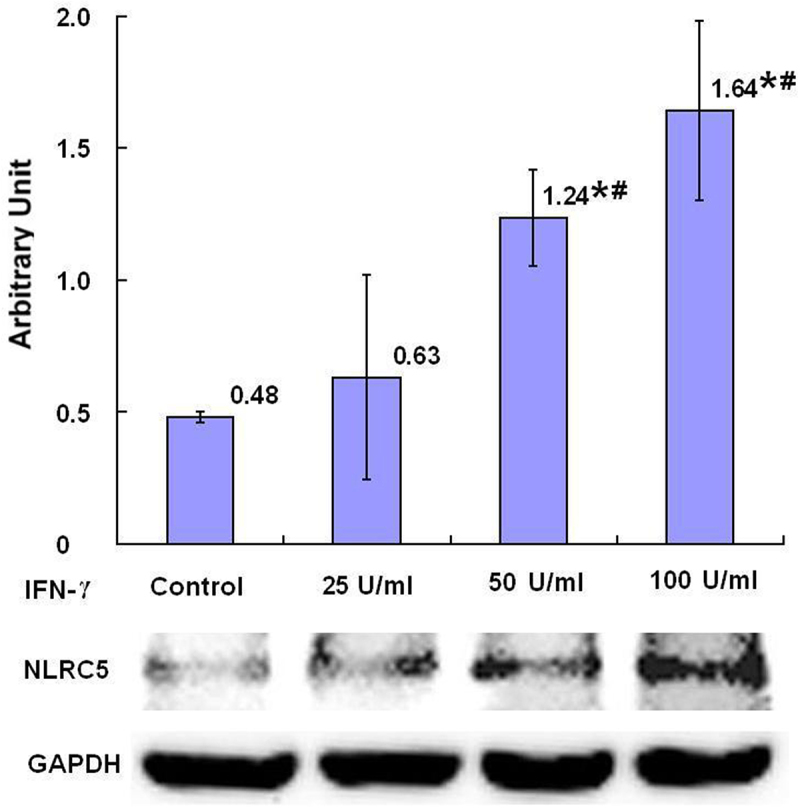
The expression of NLRC5 protein after treatment with IFN-γ (*n* = 3). After treatment of SKBR3 breast cancer cells with IFN-γ for 24 h, the expression of NLRC5 protein was detected by Western blot. Error bars indicate standard deviation of the means. Asterisks indicate LSD p-values <0.05, compared with the control after one-way anova. Hashtags indicate LSD p-values <0.05, compared with the 25 U/ml IFN-γ group after one-way anova.

The relative levels of NLRC5 in SKBR3 breast cancer cells after 24-h treatment with IFN-γ were increased in the groups treated with 50 U/ml and 100 U/ml IFN-γ, with dose dependent tendency compared with the control group with statistical significance (*p* < .05) (Figure 3). Treatment with plasma isolated from BALB/c mice after injection with IFN-γ for 24-h, the relative levels of NLRC5 in SKBR3 breast cancer cells were increased, compared with the control group (treatment with plasma isolated from BALB/c mice after injection with PBS) with statistical significance (*p* < .05) (Figure 2b).

### The expression of HLA-A, HLA-B and HLA-C mRNA and protein

**Figure 4.**
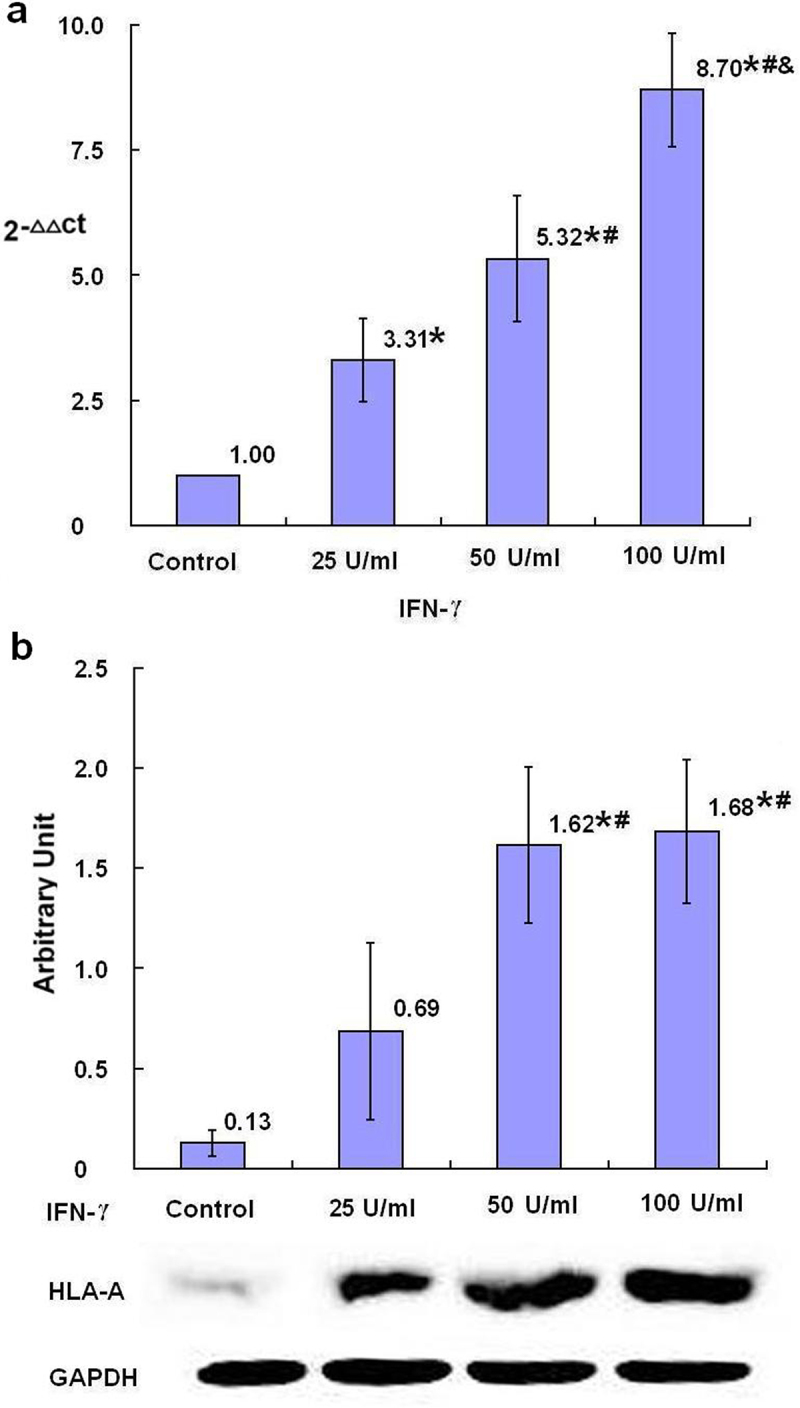
The expression of HLA-A mRNA and protein after treatment with IFN-γ (*n* = 3). After treatment of SKBR3 breast cancer cells with IFN-γ for 24 h, the expression of HLA-A mRNA and protein was detected by qRT-PCR (a) and Western blot (b), respectively. Error bars indicate standard deviation of the means. Asterisks indicate LSD p-values <0.05, compared with the control after one-way anova. Hashtags indicate LSD p-values <0.05, compared with the 25 U/ml IFN-γ group after one-way anova. Ampersands indicate LSD p-values <0.05, compared with the 50 U/ml IFN-γ group after one-way anova.

**Figure 5.**
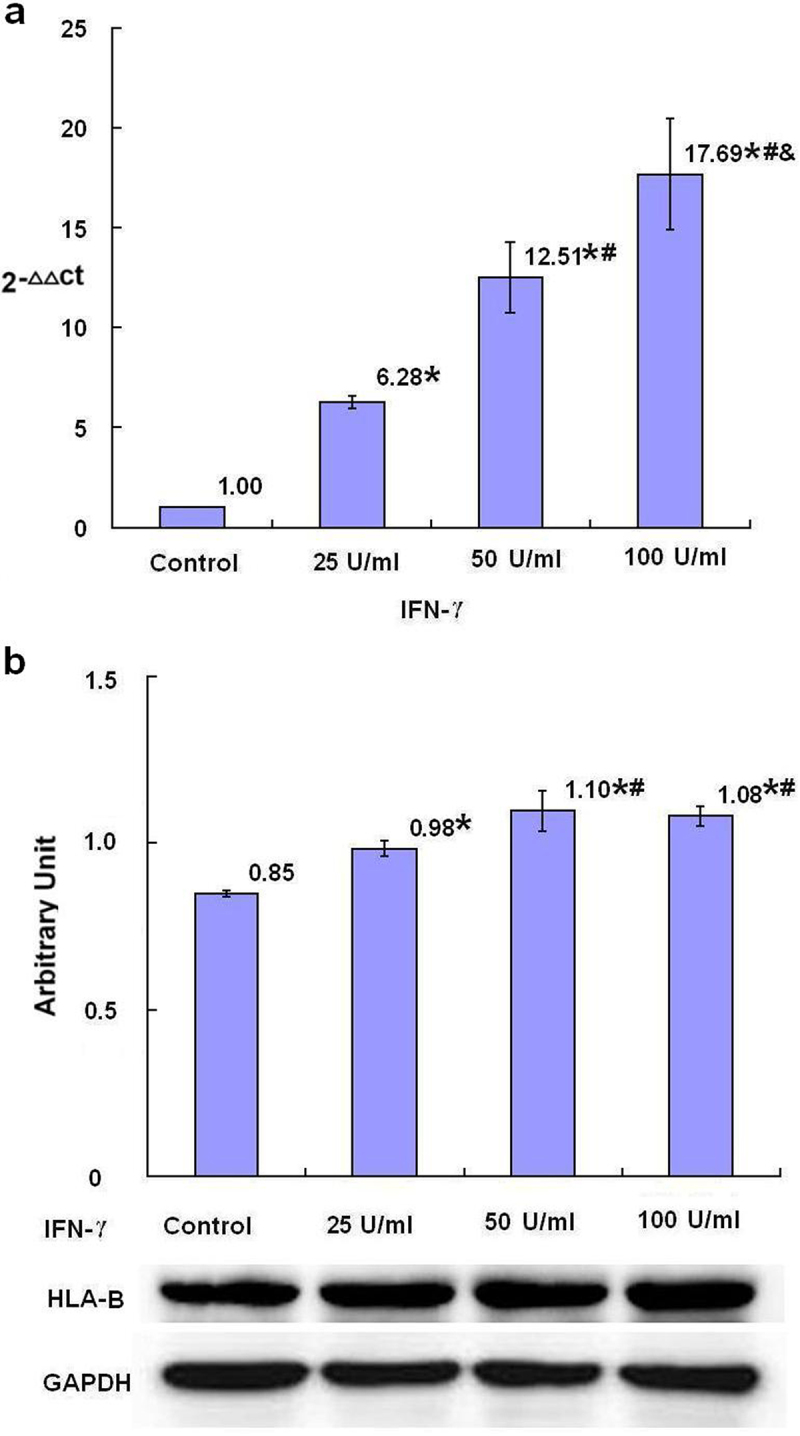
The expression of HLA-B mRNA and protein after treatment with IFN-γ (*n* = 3). After treatment of SKBR3 breast cancer cells with IFN-γ for 24 h, the expression of HLA-B mRNA and protein was detected by qRT-PCR (a) and Western blot (b), respectively. Error bars indicate standard deviation of the means. Asterisks indicate LSD p-values <0.05, compared with the control after one-way anova. Hashtags indicate LSD p-values <0.05, compared with the 25 U/ml IFN-γ group after one-way anova. Ampersands indicate LSD p-values <0.05, compared with the 50 U/ml IFN-γ group after one-way anova.

**Figure 6.**
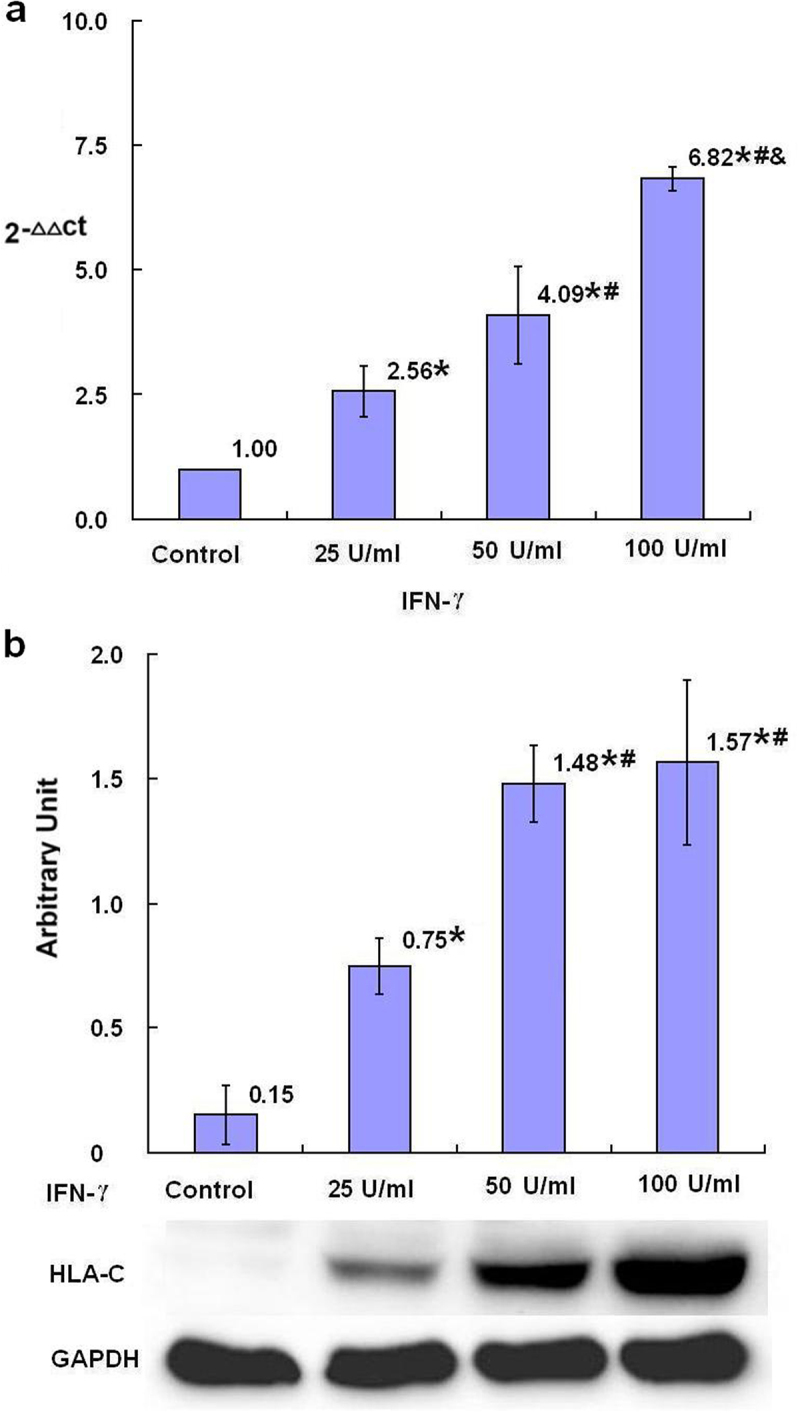
The expression of HLA-C mRNA and protein after treatment with IFN-γ (*n* = 3). After treatment of SKBR3 breast cancer cells with IFN-γ for 24 h, the expression of HLA-C mRNA and protein was detected by qRT-PCR (a) and Western blot (b), respectively. Error bars indicate standard deviation of the means. Asterisks indicate LSD p-values <0.05, compared with the control after one-way anova. Hashtags indicate LSD p-values <0.05, compared with the 25 U/ml IFN-γ group after one-way anova. Ampersands indicate LSD p-values <0.05, compared with the 50 U/ml IFN-γ group after one-way anova.

HLA-A, HLA-B and HLA-C are three classic subtypes of MHC class I molecules in human and its expression may be promoted by NLRC5. After 24-h treatment with IFN-γ, the relative levels of HLA-A, HLA-B and HLA-C mRNA in SKBR3 breast cancer cells were dose dependently increased in all groups treated with different concentrations of IFN-γ compared with the control group with statistical significance (*p* < .05) (Figures 4a, 5a and 6a). The protein levels of HLA-A in the groups treated with 50 U/ml and 100 U/ml IFN-γ, the protein levels of HLA-B and HLA-C in all groups treated with different concentrations of IFN-γ, were statistically increased with dose dependent tendency compared with the control group (*p* < .05) (Figures 4b, 5b and 6b).

### The expression of STAT1

**Figure 7.**
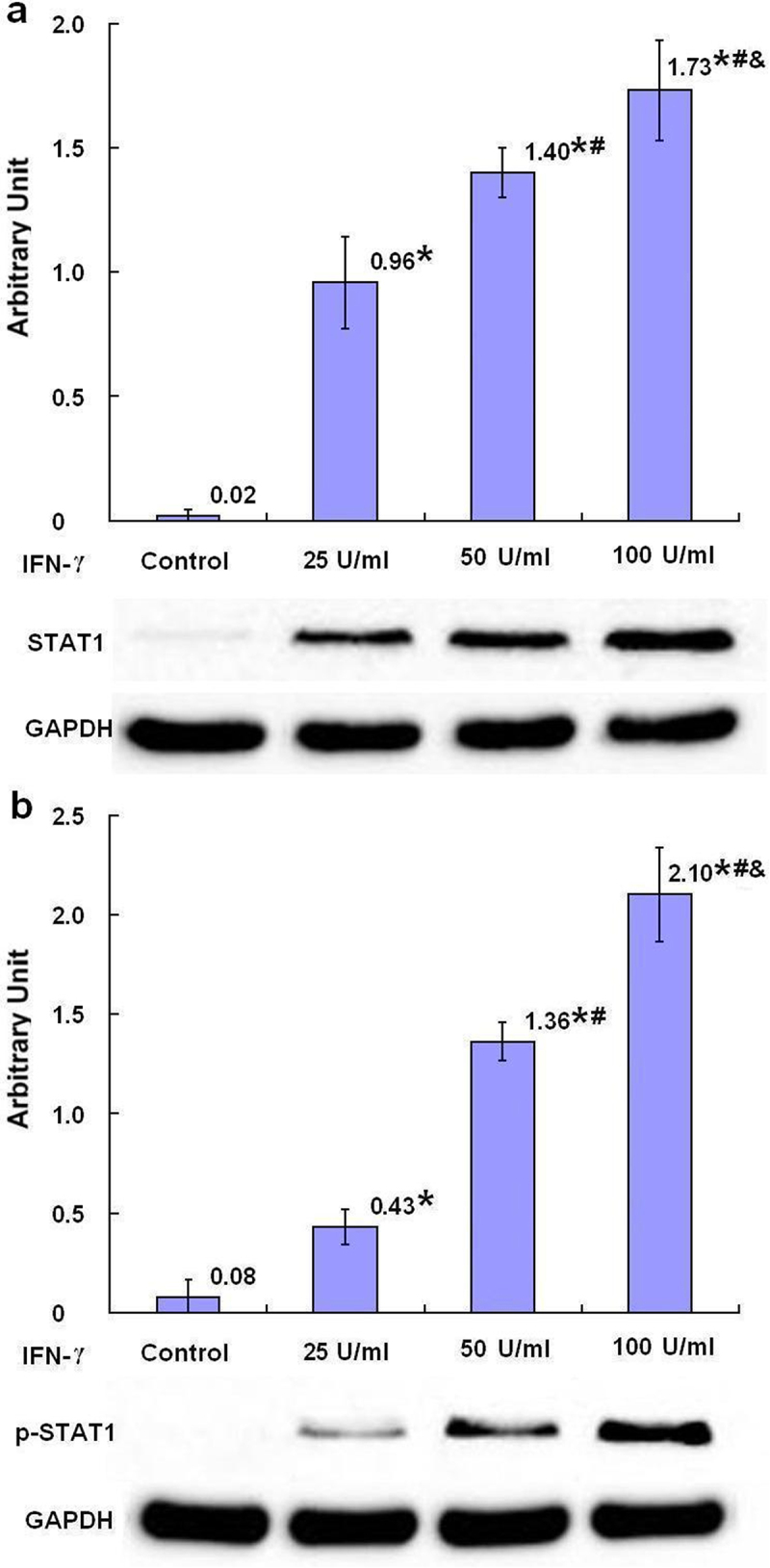
The expression of STAT1 and p-STAT1 protein after treatment with IFN-γ (*n* = 3). After treatment of SKBR3 breast cancer cells with IFN-γ for 24 h, the expression of STAT1 (a) and p-STAT1 (b) protein was detected by Western blot. Error bars indicate standard deviation of the means. Asterisks indicate LSD p-values <0.05, compared with the control after one-way anova. Hashtags indicate LSD p-values <0.05, compared with the 25 U/ml IFN-γ group after one-way anova. Ampersands indicate LSD p-values <0.05, compared with the 50 U/ml IFN-γ group after one-way anova.

Phosphorylation of STAT1 is important in JAK/STAT pathway. It was shown that, in SKBR3 breast cancer cells, after 24-h treatment with IFN-γ, the STAT1 and phosphorylated STAT1 (p-STAT1) were dose dependently increased in the groups treated with different concentrations of IFN-γ compared with the control group with statistical significance (*p* < .05) (Figure 7).

## Discussion

In traditional views, tumorigenesis is a multi-step process involving the activation of oncogenes and inactivation of tumor suppressor genes. Unlike traditional views, the mechanisms of cancer initiation and progression are not restricted to molecular alterations and instead should be considered as a complex process that interfaces with the entire organism.^27^ With its function of distinguishing non-self from self to protect the organism, the host immune system influences cancer initiation and progression. The genetic and cellular alterations in cancer enable the host immune system to generate T cell responses thereby recognizing and eradicating cancer cells.^28^ During initiation and progression, a variable number of genetic alterations and the loss of normal cellular regulatory processes accumulated in cancer. This accumulation causes the expression of neoantigens, differentiation antigens, or cancer testis antigens. The expression of these antigens results in presentation of the antigen peptides in combination with MHC class I molecules on the surface of cancer cells. The cancer-specific peptide-MHC class I complexes can be recognized by the CD8^+^ T cells, which are produced spontaneously in cancer patients, distinguished them as non-self from their normal self counterparts^29^ resulting in elimination of the cancer cells.

The microRNAs (miRNAs) exert regulative impact on innate and adaptive immune cells in both healthy and diseased conditions. In addition, miRNAs as a set of evolutionarily conserved small non-coding RNAs govern various activities and developmental stages of the immune system.^30^ The miRNAs act as post-transcriptional regulators of gene expression. The combination of complementary sequences with the 3’-UTRs of corresponding mRNA of genes encoding for proteins results in mRNA degradation or translation cessation.^31,32^ Various miRNAs play their roles in various immunological processes such as development, lineage commitment, activation, function, and aging of the immune cells.^33,34^ In addition to their impact on cellular cytokine expression, the gene expression of the entire immune system is regulated by the miRNAs involved in the activation, differentiation, and development of the T lymphocyte.^35^ The miRNAs also regulate the immunological reaction involved in cancer-associated pathways to act as oncogenes or tumor suppressors.^36,37^ MiR-4319 is a newly identified cancer-related microRNA that aberrantly expressed and became a predictor of patient survival in cancers.^38,39^ MiR-4319 exerts inhibitory effects on the proliferation of breast cancer and prostate cancer, and on cancer stemness of triple-negative breast cancer through targeting E2F2.^40,41^

How miR-4319 may intersect with broader immune escape mechanisms in breast cancer remains to be fully investigated. In colon cancer, miR-4319 is involved in regulating immune-related genes within tumor-infiltrating T cells, underscoring its potential role in shaping the immune microenvironment.^42^ Tumor associated macrophages (TAM) M2 polarization in tumor microenvironment (TME) promotes an immunosuppressive state,^43^ which contributes to cancer immune escape. In non-small cell lung cancer (NSCLC), GNAS-AS1, a long non-coding RNA, inhibits miR-4319, which promotes macrophages M2 polarization.^44^ It has been demonstrated that a direct downstream target of miR-4319 is NLRC5 which is an MHC class I transactivator (CITA). Inhibition on the miR-4319, therefore, facilitates expressional promotion on NLRC5 to repair deficient expression of MHC class I on cancer cells. Repair of the deficient MHC class I expression facilitates the induction of effective anti-cancer immunity in immunotherapy. It was shown in this study that the miR-4319 in MHC class I – deficient human SKBR3 breast cancer cells was decreased after IFN-γ treatment.

The antigen peptides either derived from endogenous proteins (self or viral), or from endocytosis of molecules, dying cells or pathogens are conventionally processed by antigen-processing pathway.^45^ The antigen-processing pathway includes proteasome-mediated degradation of the protein and transporter associated with antigen-processing (TAP)-mediated transport of the generated peptides into the endoplasmic reticulum (ER). In the ER, the peptides are loaded onto MHC class I molecules and transported to the cell surface. On the cellular surface, the peptides are displayed in combination with MHC class I molecules for MHC class I presentation. MHC class I molecules are expressed on surface of all nucleated cells. Peptides generated from proteins that are synthesized by the cell itself are presented by MHC class I molecules. CD8+ T cells are normally tolerant of these self peptides presented by the MHC class I molecules in the normal, healthy cells, but not tolerant of foreign and mutant sequences that are expressed in infected cells and in cancer cells, respectively. Such peptides are presented by MHC class I molecules as well.^45^ Efficient expression of MHC class I molecules on cancer cells are important to induce specific anticancer immune responses.

Different from MHC class II, the expression of MHC class I molecules is regulated by NLRC5. MHC class II molecules present extracellular antigenic peptides to CD4+ T cells, while MHC class I molecules present cytoplasmic antigenic peptides processed by proteasome to CD8+ T cells. In contrast to the class II transactivator (CIITA) as a master regulator of MHC class II and their accessory genes,^46^ the NLR family member NLRC5 has been identified as an MHC class I transactivator (CITA). NLRC5 specifically transactivates MHC class I genes by interacting with proximal cis regulatory elements conserved in the MHC class I promoter.^47,48^ Both CIITA and NLRC5 belong to the NLR protein family. NLRC5 is a large one in the NLR protein family and is made of 1866 amino acids in a tripartite domain structure with a molecular weight of approximately 200 kDa.^47,49–52^ The tripartite domain structure of NLRC5 contains an atypical caspase activation and recruitment domain (CARD) in the amino terminal, a nucleotide-binding domain (NBD) containing a P-loop (phosphate-binding loop) and a long leucine-rich repeats (LRRs) at the carboxy terminus.^53^ Within CARD domain, certain distinct structural features categorize it as atypical CARD, even if the CARD domain of NLRC5 consists of six alpha helices observed in the death fold domain family. NBD is essential for nucleotide binding to crucially function and localize NLRC5 in the center. Essential for nuclear import, NLRC5 also contains a nuclear localization signal (NLS).^47,54,55^ As an MHC class I transactivator, it has been revealed by gene-chip analysis that NLRC5 expression induces the expression of MHC class I and MHC class I related genes. Classical MHC class I genes (HLA-A, HLA-B, and HLA-C), non-classical MHC class I gene (HLA-E and HLA-F) and MHC class I related genes, B2M, TAP1, and PSMB9 (LMP2) are induced by NLRC5 expression. All of above NLRC5 regulated genes share similar promoter architecture as MHC class I promoters.^56^ Impairment by NLRC5 knockdown on upregulation of MHC class I expression upon IFN-γ stimulation^47^ highlights the important role of NLRC5 in both constitutive and inducible expression of MHC class I genes. NLRC5 plays a crucial role in human cancer immunity because of its importance in MHC class I expression with recruitment and activation of tumor killing CD8+ T cells. Genetic and epigenetic alterations of NLRC5 are responsible for MHC class I downregulation which is one of the important mechanisms for cancer immune escape including breast cancer.^19,57^ Promotion of NLRC5 expression facilitates host immune system to repair deficient expression of MHC class I in cancer cells. Similar to our previous study,^21^ it was shown in this study that the expression of NLRC5 in MHC class I – deficient human SKBR3 breast cancer cells was increased after IFN-γ treatment.

As a diverse set of cell surface receptors，MHC class I is expressed on all nucleated cells in the body. The protein structure of MHC class I molecules contains three alpha domain and a beta-2 macroglobulin domain.^58^ The alpha 1 and 2 domains form the binding cleft, which is flanked by tyrosine residues, to bind various peptides of cytosolic origin processed by MHC class I antigen-processing machinery. The closed ends in the binding cleft limit the size of the peptide around eight to ten amino acids to be bound, and then present them to a T cell receptor on CD8+ T cells. One end of the alpha domain also forms the binding site for an inhibitory receptor located on NK cells. The beta-2 macroglobulin plays its role in stabilizing the peptide binding. Due to the role of MHC class I in antigen presentation to CD8+ T-lymphocytes, deficient MHC class I molecules have been proved to be one of the most frequent mechanisms among the tumor escape from the host’s immunity described to date.^59,60^ Recovery of the deficient MHC class I molecules facilitates host immune system to present cancer antigens to cancer killing CD8+ T-lymphocytes for cancer elimination. Our previous study^21^ demonstrated that IFN-γ enhanced the expression of MHC class I in SKBR3 breast cancer cells. In humans, MHC also has the name HLA (human leukocyte antigen), and MHC class I has three classical subtypes, HLA-A, HLA-B, and HLA-C.^61^ In this study, it was shown that all of the three subtypes of human MHC class I (e.g. HLA-A, HLA-B, and HLA-C) were enhanced by IFN-γ with the inhibition of miR-4319 and promotion of NLRC5 expression in SKBR3 breast cancer cells.

Considering that IFN-γ is metabolized in vivo and other materials in vivo influence the effect of IFN-γ, with the failure in establishment of animal model by inoculating SKBR3 cells into Nude Mouse, the plasma isolated from the BALB/c mice after intravenous injection with IFN-γ was administrated into the SKBR3 cells in this study. It was shown that the miR-4319 was decreased and the NLRC5 was increased in MHC class I – deficient human SKBR3 breast cancer cells after treatment with the plasma.

It has been reported^3^ that IFN-γ binding to its receptor on the cellular plasma membrane causes STAT1 phosphorylation by JAK1 and JAK2. This phosphorylation followed by formation of a homodimer of STAT1 resulting in its nuclear translocation to combine with the IFN-γ activation site (GAS). This combination whereby serially induces the NLRC5 and interferon regulatory factor 1 (IRF1) and the IRF1 combines with interferon-stimulated response element (ISRE) on MHC class I genes. The nuclear translocated NLRC5 with a nuclear localization signal associates with some elements, including regulatory factor X (RFX) trimeric protein complex (RFXANK, RFXAP and RFX5), cAMP responsive element binding protein 1 (CREB1), activating transcription factor 1 (ATF1), and nuclear transcription factor Y (NF-Y), to form a complex named the class I transactivator (CITA) enhanceosome. In the CITA enhanceosome, the RFXANK, RFXAP and RFX5 bound to X1-element, CREB1 and ATF1 bound to X2-element, and NF-Y bound to Y cis-element, respectively, on the MHC class I promoters. The formation of CITA enhanceosome on the MHC class I promoters induces MHC class I gene expression. In miR-195-5p overexpressed papillary thyroid carcinoma cells and tumor xenografts, the protein levels of p-STAT1 were remarkably down-regulated but overturned by up-regulation of CircRNA NRIP1 which sponges miR-195-5p.^62^ Downregulation of miR-4319 in SKBR3 breast cancer cells by IFN-γ may curb downregulation of NLRC5 expression by miR-4319, which incommodes formation of CITA enhanceosome on the MHC class I promoters, therefore upregulates the expression of three subtypes of classic MHC class I. It was shown in this study, with the downregulation of the miR-4319 in SKBR3 breast cancer cells after 24-h treatment with IFN-γ, the STAT1 and p-STAT1 were increased, theoretically implying the involvement of JAK/STAT pathway in upregulation of NLRC-5 and three subtypes of classic MHC class I with inhibition on miR-4319 by IFN-γ in MHC-I-deficient breast cancer cells.

Beyond the JAK/STAT signaling pathway, some other pathways may also be involved in MHC-I regulation. NF-κB pathway activation upregulates β2 M and components of the antigen-processing machinery (APM).^63^ NF-κB signaling may compensate for impaired JAK/STAT activation in cases where IFN-γ fails to induce MHC-I upregulation,^64^ which causes the consideration to explore whether IFN-γ influences NF-κB activity in the context of miR-4319 suppression. Epigenetic modification, particularly histone deacetylation and DNA methylation, is another pathway involved in MHC-I regulation. Independent of IFN-γ signaling, treatment with histone deacetylase inhibitors (HDACi) or DNA methyltransferase inhibitors (DNMTi) restored MHC-I expression.^65–67^ It would therefore be significant to investigate whether IFN-γ influences miR-4319 expression through epigenetic mechanisms. Discrepantly, it has been reported that IFN-γ-induced PD-L1 expression suppressed antigen presentation and promoted immune evasion.^68^ Prolonged exposure to IFN-γ can trigger MHC-I downregulation through STAT1-mediated feedback inhibition.^69^ Therefore, further detailed investigations need to be performed in the future.

With the use of in vitro and ex vivo models, limitations arise. The use of plasma from IFN-γ-treated BALB/c mice are beneficial, but this does not fully address the lack of an in vivo tumor model, which limits the translational relevance of the findings. An appropriate breast cancer xenograft model is needed to test these mechanisms in the future. Additional negative controls such as scrambled miRNA controls in qPCR experiments would increase data reliability. A time-course would provide deeper insights into IFN-γ’s dynamic effects. Additional functional validation, such as miR-4319 overexpression or knockdown rescue experiments would benefit to establish causality between miR-4319 inhibition and NLRC5-mediated MHC-I restoration, which may be performed in the future.

## Conclusions

In summary, miR-4319 was downregulated by IFN-γ with upregulation of NLRC5 and promotion on all of the three classic subtypes of human MHC class I, HLA-A, B, and C, in SKBR3 breast cancer cells, suggesting the potential clinical relevance on counteracting cancer evasion from immunosurveillance and benefiting cancer immunotherapy. It is significant to clarify whether similar effects may occur in other MHC-I-deficient cancers. Limitations exist in this study such as the absence of in vivo validation and functional rescue experiments.

## Supplementary Material

renamed_a7d9c.docx

fig S6 p ATAT1.TIF

fig S3 HLA B.TIF

fig S5 ATAT1.TIF

fig S1 NLRC5.TIF

fig S4 HLA C.TIF

fig S2 HLA A.TIF

## Data Availability

All the data generated or analyzed during this study are included in this published article.
