# Supplementary material for: IFN-γ downregulates miR-4319 to enhance NLRC5 and MHC-I expression in MHC-I-deficient breast cancer cells
Source: Cancer Biol Ther. 2025 Jul 1;26(1):2523621. doi: 10.1080/15384047.2025.2523621 (PMC12258810; doi:10.1080/15384047.2025.2523621)
Supplement: renamed_a7d9c.docx [file KCBT_A_2523621_SM1248.docx]

Fig S1. The full western blot image of NLRC5 protein expression without cropping after treatment with IFN-γ detected by supplementary western blot assay. After treatment of SKBR3 breast cancer cells with IFN-γ for 24h, the expression of NLRC5 protein was detected by Western blot.

Alt-text:

A western blot image (a) with five electrophoretic lanes with the protein bands which represent the protein marker, the control group, the 25 U/ml IFN-γ group, the 50 U/ml IFN-γ group and the 100 U/ml IFN-γ group, respectively, displays the NLRC5 protein expression levels after treatment of SKBR3 breast cancer cells with IFN-γ for 24h; a western blot image (b) with five electrophoretic lanes with the protein bands which represent the protein marker, the control group, the 25 U/ml IFN-γ group, the 50 U/ml IFN-γ group and the 100 U/ml IFN-γ group, respectively, displays the GAPDH protein expression levels after treatment of SKBR3 breast cancer cells with IFN-γ for 24h.

Fig S2. The full western blot image of HLA-A protein expression without cropping after treatment with IFN-γ detected by supplementary western blot assay. After treatment of SKBR3 breast cancer cells with IFN-γ for 24h, the expression of HLA-A protein was detected by Western blot.

Alt-text:

A western blot image with five electrophoretic lanes with the protein bands which represent the protein marker, the control group, the 25 U/ml IFN-γ group, the 50 U/ml IFN-γ group and the 100 U/ml IFN-γ group, respectively, displays the HLA-A protein expression levels after treatment of SKBR3 breast cancer cells with IFN-γ for 24h; a western blot image (b) with five electrophoretic lanes with the protein bands which represent the protein marker, the control group, the 25 U/ml IFN-γ group, the 50 U/ml IFN-γ group and the 100 U/ml IFN-γ group, respectively, displays the GAPDH protein expression levels after treatment of SKBR3 breast cancer cells with IFN-γ for 24h.

Fig S3. The full western blot image of HLA-B protein expression without cropping after treatment with IFN-γ detected by supplementary western blot assay. After treatment of SKBR3 breast cancer cells with IFN-γ for 24h, the expression of HLA-B protein was detected by Western blot.

Alt-text:

A western blot image with five electrophoretic lanes with the protein bands which represent the protein marker, the control group, the 25 U/ml IFN-γ group, the 50 U/ml IFN-γ group and the 100 U/ml IFN-γ group, respectively, displays the HLA-B protein expression levels after treatment of SKBR3 breast cancer cells with IFN-γ for 24h; a western blot image (b) with five electrophoretic lanes with the protein bands which represent the protein marker, the control group, the 25 U/ml IFN-γ group, the 50 U/ml IFN-γ group and the 100 U/ml IFN-γ group, respectively, displays the GAPDH protein expression levels after treatment of SKBR3 breast cancer cells with IFN-γ for 24h.

Fig S4. The full western blot image of HLA-C protein expression without cropping after treatment with IFN-γ detected by supplementary western blot assay. After treatment of SKBR3 breast cancer cells with IFN-γ for 24h, the expression of HLA-C protein was detected by Western blot.

Alt-text:

A western blot image with five electrophoretic lanes with the protein bands which represent the protein marker, the control group, the 25 U/ml IFN-γ group, the 50 U/ml IFN-γ group and the 100 U/ml IFN-γ group, respectively, displays the HLA-C protein expression levels after treatment of SKBR3 breast cancer cells with IFN-γ for 24h; a western blot image (b) with five electrophoretic lanes with the protein bands which represent the protein marker, the control group, the 25 U/ml IFN-γ group, the 50 U/ml IFN-γ group and the 100 U/ml IFN-γ group, respectively, displays the GAPDH protein expression levels after treatment of SKBR3 breast cancer cells with IFN-γ for 24h.

Fig S5. The full western blot image of STAT1 protein expression without cropping after treatment with IFN-γ detected by supplementary western blot assay. After treatment of SKBR3 breast cancer cells with IFN-γ for 24h, the expression of STAT1 protein was detected by Western blot.

Alt-text:

A western blot image with five electrophoretic lanes with the protein bands which represent the protein marker, the control group, the 25 U/ml IFN-γ group, the 50 U/ml IFN-γ group and the 100 U/ml IFN-γ group, respectively, displays the STAT1 protein expression levels after treatment of SKBR3 breast cancer cells with IFN-γ for 24h; a western blot image (b) with five electrophoretic lanes with the protein bands which represent the protein marker, the control group, the 25 U/ml IFN-γ group, the 50 U/ml IFN-γ group and the 100 U/ml IFN-γ group, respectively, displays the GAPDH protein expression levels after treatment of SKBR3 breast cancer cells with IFN-γ for 24h.

Fig S6. The full western blot image of p-STAT1 protein expression without cropping after treatment with IFN-γ detected by supplementary western blot assay. After treatment of SKBR3 breast cancer cells with IFN-γ for 24h, the expression of p-STAT1 protein was detected by Western blot.

Alt-text:

A western blot image with five electrophoretic lanes with the protein bands which represent the protein marker, the control group, the 25 U/ml IFN-γ group, the 50 U/ml IFN-γ group and the 100 U/ml IFN-γ group, respectively, displays the p-STAT1 protein expression levels after treatment of SKBR3 breast cancer cells with IFN-γ for 24h; a western blot image (b) with five electrophoretic lanes with the protein bands which represent the protein marker, the control group, the 25 U/ml IFN-γ group, the 50 U/ml IFN-γ group and the 100 U/ml IFN-γ group, respectively, displays the GAPDH protein expression levels after treatment of SKBR3 breast cancer cells with IFN-γ for 24h.
