# Supplementary figures and images for: IFN-γ downregulates miR-4319 to enhance NLRC5 and MHC-I expression in MHC-I-deficient breast cancer cells
Source: Cancer Biol Ther. 2025 Jul 1;26(1):2523621. doi: 10.1080/15384047.2025.2523621 (PMC12258810; doi:10.1080/15384047.2025.2523621)

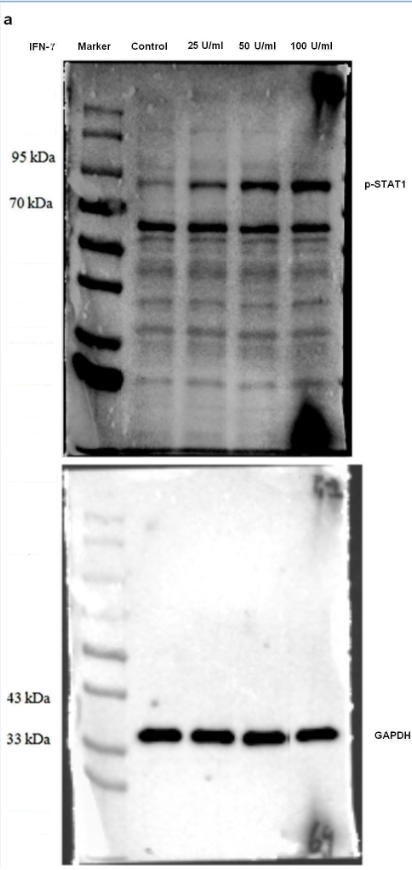

Supplement: fig S6 p ATAT1.TIF [file KCBT_A_2523621_SM1247.tif]

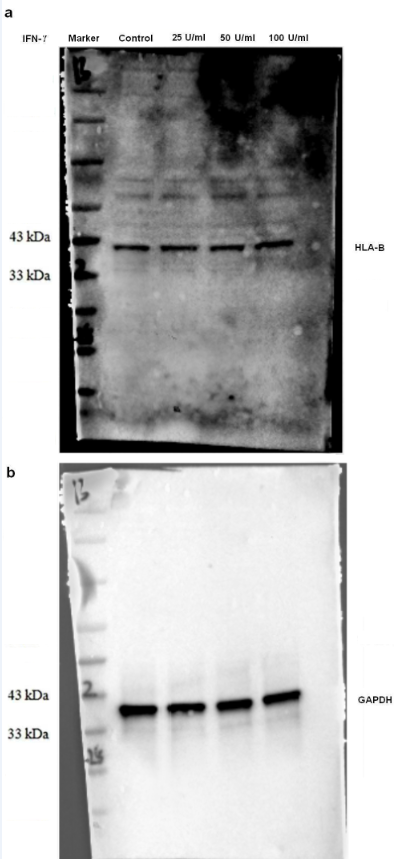

Supplement: fig S3 HLA B.TIF [file KCBT_A_2523621_SM1246.tif]

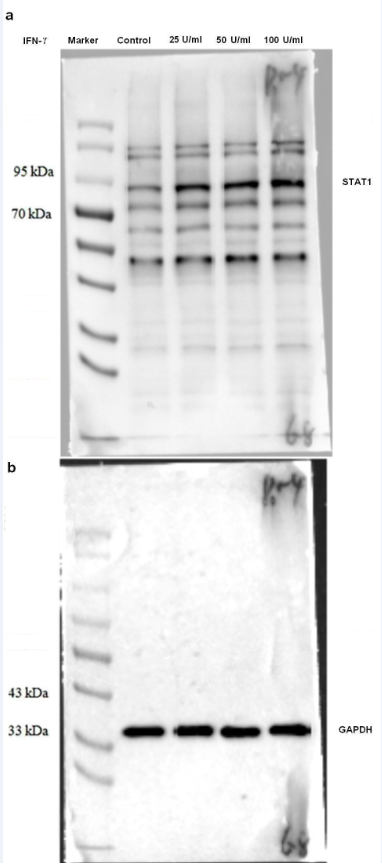

Supplement: fig S5 ATAT1.TIF [file KCBT_A_2523621_SM1245.tif]

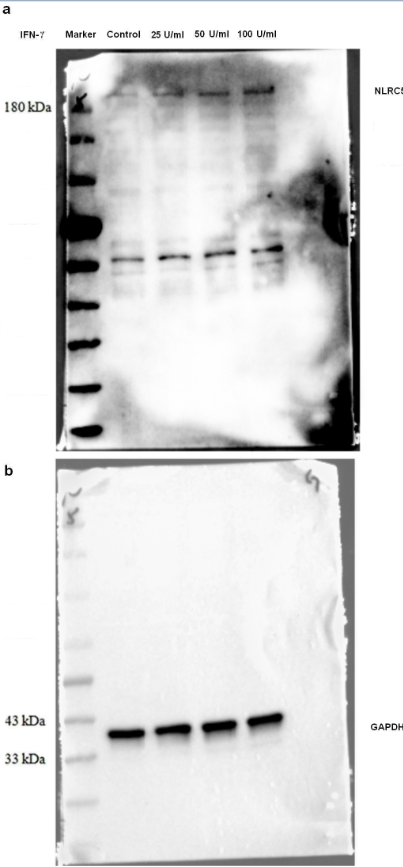

Supplement: fig S1 NLRC5.TIF [file KCBT_A_2523621_SM1244.tif]

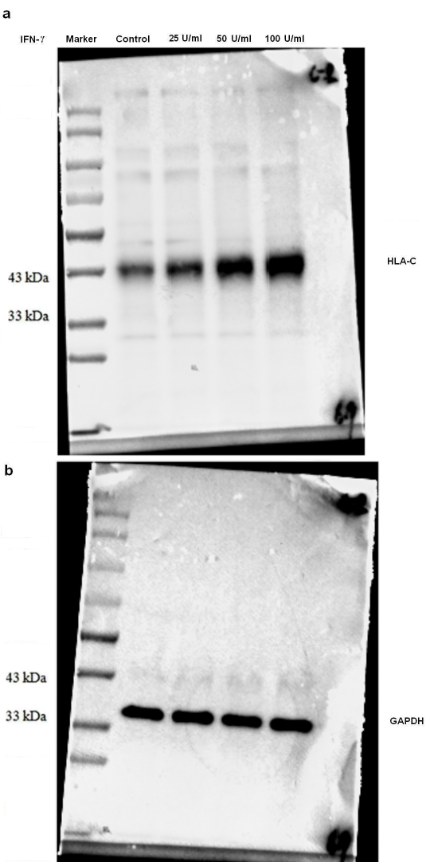

Supplement: fig S4 HLA C.TIF [file KCBT_A_2523621_SM1243.tif]

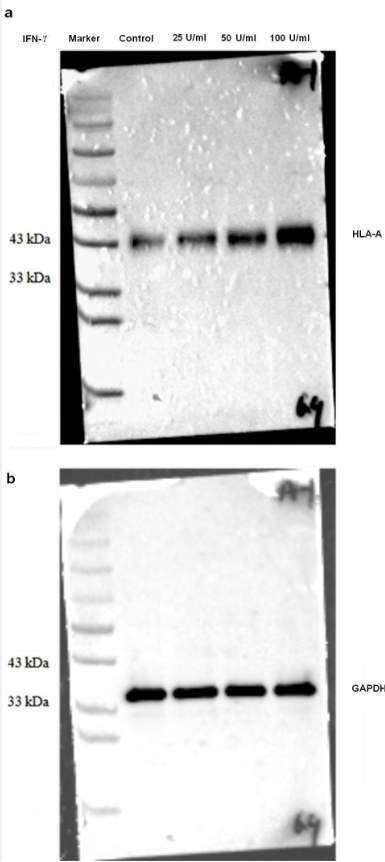

Supplement: fig S2 HLA A.TIF [file KCBT_A_2523621_SM1242.tif]
